# Aqua{4,4′,6,6′-tetrachloro-2,2′-[(2,2-dimethylpropane-1,3-diyl)bis(nitrilomethanylylidene)]diphenolato}zinc

**DOI:** 10.1107/S1600536812025986

**Published:** 2012-06-16

**Authors:** Hadi Kargar, Reza Kia, Saeideh Abbasian, Muhammad Nawaz Tahir

**Affiliations:** a Department of Chemistry, Payame Noor University, PO BOX 19395-3697 Tehran, I. R. IRAN; b Department of Chemistry, Science and Research Branch, Islamic Azad University, Tehran, Iran; c Department of Physics, University of Sargodha, Punjab, Pakistan

## Abstract

The asymmetric unit of the title compound, [Zn(C_19_H_16_Cl_4_N_2_O_2_)(H_2_O)], comprises two crystallographically independent mol­ecules. The geometry around the Zn^II^ atoms is distorted trigonal–bipyramidal, supported by the N_2_O_2_ donor atoms of the tetradentate Schiff base and a coordinating water mol­ecule. The dihedral angles between the benzene rings in the two mol­ecules are 34.10 (15) Å and 30.61 (15) Å. In the crystal, neighbouring independent mol­ecules are linked by pairs of O—H⋯O hydrogen bonds, forming dimers with *R* ^2^_2_(6) ring motifs, and by O—H⋯Cl hydrogen bonds. There are short Cl⋯Cl [3.4728 (16), 3.4863 (16), and 3.388 (1) Å] contacts present, and mol­ecules are also linked by C—H⋯O and π–π [centroid–centroid distance = 3.671 (2) Å] inter­actions.

## Related literature

For applications of Schiff base ligands in coordination chemistry, see: Granovski *et al.* (1993 ▶); Blower *et al.* (1998 ▶). For a related structure, see: Zhong-Lu *et al.* (2006 ▶). For standard bond lengths, see: Allen *et al.* (1987 ▶). For hydrogen-bond motifs, see: Bernstein *et al.* (1995 ▶). For van der Waals radii, see: Bondi (1964 ▶).
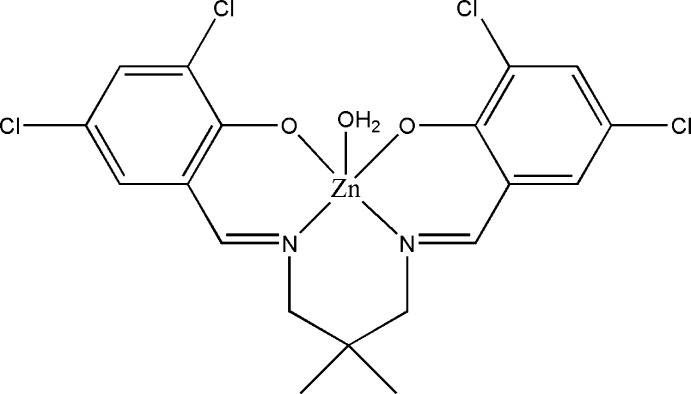


## Experimental

### 

#### Crystal data

[Zn(C_19_H_16_Cl_4_N_2_O_2_)(H_2_O)]
*M* *_r_* = 529.52
Monoclinic, 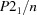

*a* = 11.2812 (7) Å
*b* = 22.5897 (15) Å
*c* = 17.6777 (12) Å
β = 107.159 (3)°
*V* = 4304.4 (5) Å^3^
*Z* = 8
Mo *K*α radiation
μ = 1.66 mm^−1^
*T* = 291 K
0.35 × 0.20 × 0.18 mm

#### Data collection

Bruker SMART APEXII CCD area-detector diffractometer
Absorption correction: multi-scan (*SADABS*; Bruker, 2005 ▶) *T*_min_ = 0.594, *T*_max_ = 0.754
40205 measured reflections
10321 independent reflections
6266 reflections with *I* > 2σ(*I*)
*R*_int_ = 0.053

#### Refinement

*R*[*F* ^2^ > 2σ(*F* ^2^)] = 0.047
*wR*(*F* ^2^) = 0.094
*S* = 1.00
10321 reflections
525 parameters
H-atom parameters constrained
Δρ_max_ = 0.54 e Å^−3^
Δρ_min_ = −0.55 e Å^−3^

### 

Data collection: *APEX2* (Bruker, 2005 ▶); cell refinement: *SAINT* (Bruker, 2005 ▶); data reduction: *SAINT*; program(s) used to solve structure: *SHELXS97* (Sheldrick, 2008 ▶); program(s) used to refine structure: *SHELXL97* (Sheldrick, 2008 ▶); molecular graphics: *SHELXTL* (Sheldrick, 2008 ▶); software used to prepare material for publication: *SHELXTL* and *PLATON* (Spek, 2009 ▶).

## Supplementary Material

Crystal structure: contains datablock(s) global, I. DOI: 10.1107/S1600536812025986/su2451sup1.cif

Structure factors: contains datablock(s) I. DOI: 10.1107/S1600536812025986/su2451Isup2.hkl

Additional supplementary materials:  crystallographic information; 3D view; checkCIF report

## Figures and Tables

**Table 1:** Hydrogen-bond geometry (Å, °)

*D*—H⋯*A*	*D*—H	H⋯*A*	*D*⋯*A*	*D*—H⋯*A*
O1*W*—H1*W*1⋯Cl5^i^	0.89	2.76	3.472 (2)	139
O1*W*—H1*W*1⋯O3^i^	0.89	2.05	2.825 (3)	145
O1*W*—H2*W*1⋯Cl8^i^	0.89	2.62	3.235 (2)	127
O1*W*—H2*W*1⋯O4^i^	0.89	1.86	2.681 (3)	153
O2*W*—H1*W*2⋯Cl4^ii^	0.88	2.51	3.226 (2)	139
O2*W*—H1*W*2⋯O2^ii^	0.88	2.04	2.807 (3)	144
O2*W*—H2*W*2⋯Cl1^ii^	0.89	2.62	3.340 (2)	139
O2*W*—H2*W*2⋯O1^ii^	0.89	2.01	2.749 (3)	140
C8—H8*A*⋯O4^iii^	0.97	2.56	3.310 (4)	134

Symmetry codes: (i) 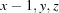; (ii) 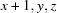; (iii) 
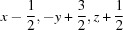
.

**Fig. 1.**
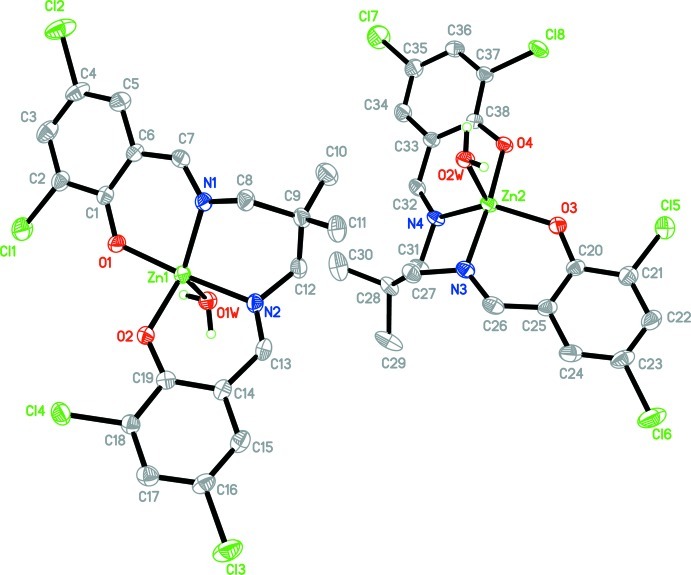
The molecular structure of the title compound, showing 40% probability displacement ellipsoids and the atom numbering. The C-bound H atoms have been omitted for clarity.

**Fig. 2.**
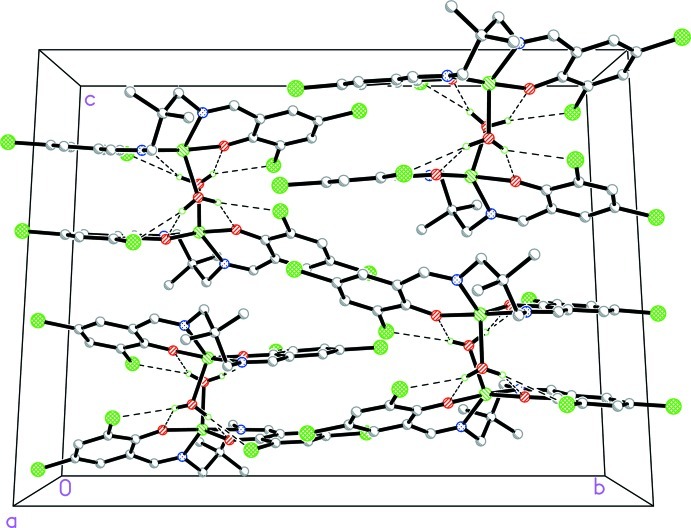
The crystal packing of the title compound viewed down the a-axis, showing linking of individual dimers formed via O—H···O hydrogen bonds (dashed lines). The hydrogen atoms not involved in these interactions have been omitted for clarity.

**Fig. 3.**
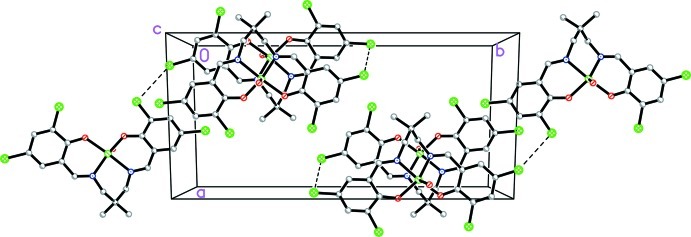
The packing diagram of the title compound viewed down the c-axis, showing the Cl···Cl interactions (dashed lines). The hydrogen atoms have been omitted for clarity.

## supplementary crystallographic information

### Comment

Schiff base complexes are one of the most important stereochemical models in transition metal coordination chemistry, with their ease of preparation and structural variations (Granovski *et al.*, 1993; Blower *et al.*, (1998).

The asymmetric unit of the title compound, Fig. 1, comprises two crystallographically independent molecules. The bond lengths (Allen *et al.*, 1987) and angles are within the normal ranges and are comparable to those found for a related structure (Zhong-Lu *et al.* 2006). The geometry around the Zn^II^ atom is a distorted trigonal-bipyramide which is supported by the N_2_O_2_ donor atoms of the coordinated Schiff base and a coordinated water molecule. The dihedral angles between the benzene rings are 34.10 (15) Å [C1-C6/C14-C19] and 30.61 (15) Å [C20-C25/C33-C38].

In the crystal, neighbouring independent molecules are linked by pairs of O—H···O hydrogen bonds forming dimers with *R^2^_2_(6)* ring motifs (Bernstein *et al.*, 1995), and by O-H···Cl hydrogen bonds (Table 1 and Fig. 2). Short Cl···Cl [Cl2···Cl6^iv^ = 3.4728 (16)Å, (iv) x-1/2, -y+3/2, z+1/2; Cl4···Cl7^v^ = 3.4863 (16)Å, (v) -x+1/2, y-1/2, -z+3/2; Cl6···Cl8^vi^ = 3.388 (1)Å, (vi) -x+5/2, y-1/2, -z+3/2] contacts are present in the crystal structure (Fig. 3); they are shorter than the sum of the van der Waals radii of Cl atoms [3.50 Å; Bondi, 1964]. The crystal structure is further stabilized C—H···O (Table 1) and π···π interactions [*Cg*1···*Cg*2^i^ = 3.671 (2)Å, (i) x-1, y, z; *Cg*1 and *Cg*2 are the centroids of the C14–C19 and C20–C25 benzene rings, respectively].

### Experimental

The title compound was synthesized by adding 3,5-dichloro-salicylaldehyde-2,2-dimethyl-1,3- propanediamine (2 mmol) to a solution of Zn(OAc)_2_. 2H_2_O (2.1 mmol) in ethanol (30 ml). The mixture was refluxed with stirring for half an hour. The resultant solution was filtered. Light-green single crystals of the title compound, suitable for *X*-ray structure determination, were recrystallized from ethanol by slow evaporation of the solvents at room temperature over several days.

### Refinement

The H atoms of the water molecules were located in a difference Fourier map and were constrained to ride on the parent O atoms, with U_iso_(H) = 1.5U_eq_(O). The C-bound H-atoms were included in calculated positions and treated as riding atoms: C-H = 0.93, 0.96 and 0.97 Å for CH, CH_3_ and CH_2_ H-atoms, respectively, with U_iso_(H) = k × U_eq_(parent C-atom), where k = 1.5 for CH_3_ H-atoms and = 1.2 for other H-atoms.

### Crystal data

**Table:**

[Zn(C_19_H_16_Cl_4_N_2_O_2_)(H_2_O)]	*F*(000) = 2144
*M**_r_* = 529.52	*D*_x_ = 1.634 Mg m^−^^3^
Monoclinic, *P*2_1_/*n*	Mo *K*α radiation, λ = 0.71073 Å
Hall symbol: -P 2yn	Cell parameters from 3535 reflections
*a* = 11.2812 (7) Å	θ = 2.5–27.5°
*b* = 22.5897 (15) Å	µ = 1.66 mm^−^^1^
*c* = 17.6777 (12) Å	*T* = 291 K
β = 107.159 (3)°	Block, light-green
*V* = 4304.4 (5) Å^3^	0.35 × 0.20 × 0.18 mm
*Z* = 8	

### Data collection

**Table:**

Bruker SMART APEXII CCD area-detector diffractometer	10321 independent reflections
Radiation source: fine-focus sealed tube	6266 reflections with *I* > 2σ(*I*)
Graphite monochromator	*R*_int_ = 0.053
φ and ω scans	θ_max_ = 28.0°, θ_min_ = 1.8°
Absorption correction: multi-scan (*SADABS*; Bruker, 2005)	*h* = −14→14
*T*_min_ = 0.594, *T*_max_ = 0.754	*k* = −27→29
40205 measured reflections	*l* = −13→23

### Refinement

**Table:**

Refinement on *F*^2^	Primary atom site location: structure-invariant direct methods
Least-squares matrix: full	Secondary atom site location: difference Fourier map
*R*[*F*^2^ > 2σ(*F*^2^)] = 0.047	Hydrogen site location: inferred from neighbouring sites
*wR*(*F*^2^) = 0.094	H-atom parameters constrained
*S* = 1.00	*w* = 1/[σ^2^(*F*_o_^2^) + (0.0289*P*)^2^ + 2.1802*P*] where *P* = (*F*_o_^2^ + 2*F*_c_^2^)/3
10321 reflections	(Δ/σ)_max_ = 0.001
525 parameters	Δρ_max_ = 0.54 e Å^−^^3^
0 restraints	Δρ_min_ = −0.55 e Å^−^^3^

### Special details

**Table:**

Geometry. All esds (except the esd in the dihedral angle between two l.s. planes) are estimated using the full covariance matrix. The cell esds are taken into account individually in the estimation of esds in distances, angles and torsion angles; correlations between esds in cell parameters are only used when they are defined by crystal symmetry. An approximate (isotropic) treatment of cell esds is used for estimating esds involving l.s. planes.
Refinement. Refinement of F^2^ against ALL reflections. The weighted R-factor wR and goodness of fit S are based on F^2^, conventional R-factors R are based on F, with F set to zero for negative F^2^. The threshold expression of F^2^ > 2sigma(F^2^) is used only for calculating R-factors(gt) etc. and is not relevant to the choice of reflections for refinement. R-factors based on F^2^ are statistically about twice as large as those based on F, and R- factors based on ALL data will be even larger.

### Fractional atomic coordinates and isotropic or equivalent isotropic displacement parameters (Å^2^)

**Table:**

	*x*	*y*	*z*	*U*_iso_*/*U*_eq_	
C1	0.1705 (3)	0.87606 (14)	0.92060 (17)	0.0355 (7)	
C2	0.0832 (3)	0.92301 (15)	0.90671 (18)	0.0437 (8)	
C3	0.1138 (3)	0.97980 (16)	0.93028 (19)	0.0537 (10)	
H3	0.0532	1.0091	0.9192	0.064*	
C4	0.2353 (4)	0.99363 (14)	0.97075 (19)	0.0506 (9)	
C5	0.3224 (3)	0.95054 (15)	0.98837 (19)	0.0480 (9)	
H5	0.4034	0.9601	1.0170	0.058*	
C6	0.2935 (3)	0.89209 (14)	0.96451 (17)	0.0372 (8)	
C7	0.3924 (3)	0.84976 (15)	0.99221 (17)	0.0391 (8)	
H7	0.4661	0.8643	1.0263	0.047*	
C8	0.4941 (3)	0.75817 (14)	1.02127 (18)	0.0415 (8)	
H8A	0.4612	0.7280	1.0485	0.050*	
H8B	0.5496	0.7828	1.0613	0.050*	
C9	0.5697 (3)	0.72769 (14)	0.97352 (18)	0.0382 (8)	
C10	0.6560 (3)	0.77180 (17)	0.9508 (2)	0.0583 (10)	
H10A	0.7135	0.7875	0.9980	0.087*	
H10B	0.6078	0.8035	0.9207	0.087*	
H10C	0.7008	0.7521	0.9196	0.087*	
C11	0.6452 (3)	0.67965 (16)	1.0274 (2)	0.0540 (10)	
H11A	0.6891	0.6967	1.0777	0.081*	
H11B	0.7034	0.6628	1.0034	0.081*	
H11C	0.5904	0.6493	1.0352	0.081*	
C12	0.4866 (3)	0.69988 (15)	0.89747 (18)	0.0406 (8)	
H12A	0.4726	0.7285	0.8548	0.049*	
H12B	0.5289	0.6661	0.8835	0.049*	
C13	0.3407 (3)	0.62610 (15)	0.90478 (18)	0.0404 (8)	
H13	0.4012	0.5992	0.9013	0.048*	
C14	0.2239 (3)	0.60225 (14)	0.90957 (17)	0.0373 (8)	
C15	0.2108 (3)	0.54062 (15)	0.90541 (18)	0.0446 (8)	
H15	0.2760	0.5174	0.9002	0.054*	
C16	0.1047 (3)	0.51410 (14)	0.90886 (19)	0.0454 (9)	
C17	0.0072 (3)	0.54684 (15)	0.91738 (18)	0.0446 (8)	
H17	−0.0652	0.5285	0.9201	0.053*	
C18	0.0189 (3)	0.60743 (14)	0.92176 (17)	0.0366 (7)	
C19	0.1247 (3)	0.63827 (14)	0.91680 (16)	0.0336 (7)	
C20	1.0088 (3)	0.61283 (14)	0.70433 (17)	0.0372 (8)	
C21	1.1082 (3)	0.57707 (16)	0.69715 (19)	0.0466 (9)	
C22	1.1025 (4)	0.51679 (16)	0.6906 (2)	0.0546 (10)	
H22	1.1699	0.4954	0.6851	0.065*	
C23	0.9963 (4)	0.48839 (15)	0.6923 (2)	0.0570 (11)	
C24	0.8978 (3)	0.51962 (16)	0.70139 (19)	0.0526 (10)	
H24	0.8270	0.4995	0.7036	0.063*	
C25	0.9019 (3)	0.58158 (14)	0.70739 (18)	0.0417 (8)	
C26	0.7934 (3)	0.61027 (16)	0.71670 (18)	0.0446 (9)	
H26	0.7299	0.5859	0.7219	0.054*	
C27	0.6590 (3)	0.68856 (16)	0.72515 (19)	0.0454 (9)	
H27A	0.6754	0.7213	0.7624	0.055*	
H27B	0.6174	0.6577	0.7459	0.055*	
C28	0.5728 (3)	0.70994 (16)	0.6448 (2)	0.0475 (9)	
C29	0.4962 (4)	0.65769 (18)	0.6003 (2)	0.0755 (13)	
H29A	0.4435	0.6430	0.6299	0.113*	
H29B	0.5510	0.6268	0.5942	0.113*	
H29C	0.4462	0.6706	0.5491	0.113*	
C30	0.4885 (4)	0.7576 (2)	0.6605 (2)	0.0791 (14)	
H30A	0.4322	0.7706	0.6112	0.119*	
H30B	0.5377	0.7905	0.6865	0.119*	
H30C	0.4423	0.7420	0.6937	0.119*	
C31	0.6459 (3)	0.73261 (15)	0.59062 (18)	0.0421 (8)	
H31A	0.6770	0.6989	0.5683	0.051*	
H31B	0.5899	0.7542	0.5472	0.051*	
C32	0.7426 (3)	0.82606 (15)	0.61298 (17)	0.0375 (8)	
H32	0.6662	0.8394	0.5811	0.045*	
C33	0.8389 (3)	0.86968 (13)	0.63842 (17)	0.0356 (7)	
C34	0.8040 (3)	0.92845 (15)	0.6206 (2)	0.0477 (9)	
H34A	0.7213	0.9374	0.5953	0.057*	
C35	0.8887 (3)	0.97289 (15)	0.6396 (2)	0.0542 (10)	
C36	1.0125 (3)	0.96035 (15)	0.6740 (2)	0.0476 (9)	
H36	1.0707	0.9908	0.6861	0.057*	
C37	1.0490 (3)	0.90312 (14)	0.69004 (18)	0.0378 (8)	
C38	0.9654 (3)	0.85496 (14)	0.67545 (16)	0.0318 (7)	
Cl1	−0.06983 (8)	0.90631 (4)	0.85779 (6)	0.0657 (3)	
Cl2	0.27402 (11)	1.06605 (4)	1.00096 (6)	0.0797 (4)	
Cl3	0.08933 (11)	0.43764 (4)	0.90066 (6)	0.0712 (3)	
Cl4	−0.10329 (8)	0.64954 (4)	0.93279 (5)	0.0506 (2)	
Cl5	1.24528 (9)	0.61189 (5)	0.69905 (6)	0.0663 (3)	
Cl6	0.98712 (11)	0.41141 (4)	0.68558 (7)	0.0866 (4)	
Cl7	0.84276 (11)	1.04590 (5)	0.61807 (9)	0.0951 (4)	
Cl8	1.20501 (7)	0.88856 (4)	0.73122 (5)	0.0536 (2)	
N1	0.3909 (2)	0.79496 (12)	0.97557 (14)	0.0346 (6)	
N2	0.3672 (2)	0.68069 (12)	0.90498 (14)	0.0354 (6)	
N3	0.7758 (2)	0.66583 (12)	0.71855 (14)	0.0378 (6)	
N4	0.7508 (2)	0.77119 (12)	0.62946 (14)	0.0347 (6)	
O1	0.13710 (18)	0.82302 (9)	0.89589 (12)	0.0412 (5)	
O2	0.12543 (18)	0.69585 (9)	0.91997 (12)	0.0376 (5)	
O3	1.02176 (19)	0.67011 (9)	0.70831 (12)	0.0410 (5)	
O4	1.00460 (17)	0.80174 (9)	0.69400 (12)	0.0370 (5)	
O1W	0.21606 (18)	0.74985 (9)	0.77822 (11)	0.0392 (5)	
H1W1	0.1832	0.7147	0.7622	0.059*	
H2W1	0.1602	0.7775	0.7558	0.059*	
O2W	0.92427 (18)	0.76063 (9)	0.82454 (11)	0.0394 (5)	
H1W2	0.9594	0.7313	0.8563	0.059*	
H2W2	0.9672	0.7942	0.8352	0.059*	
Zn1	0.24468 (3)	0.751893 (16)	0.89909 (2)	0.03454 (10)	
Zn2	0.90046 (3)	0.732396 (16)	0.71064 (2)	0.03484 (10)	

### Atomic displacement parameters (Å^2^)

**Table:**

	*U*^11^	*U*^22^	*U*^33^	*U*^12^	*U*^13^	*U*^23^
C1	0.0374 (18)	0.036 (2)	0.0333 (16)	0.0063 (15)	0.0103 (14)	−0.0003 (14)
C2	0.0412 (19)	0.044 (2)	0.0424 (19)	0.0104 (16)	0.0074 (15)	−0.0026 (15)
C3	0.065 (3)	0.043 (2)	0.049 (2)	0.0189 (19)	0.0093 (19)	−0.0014 (17)
C4	0.073 (3)	0.030 (2)	0.043 (2)	0.0067 (19)	0.0067 (18)	−0.0041 (15)
C5	0.053 (2)	0.046 (2)	0.043 (2)	−0.0013 (18)	0.0105 (17)	−0.0055 (16)
C6	0.0409 (18)	0.0335 (19)	0.0378 (17)	0.0029 (15)	0.0126 (14)	−0.0043 (14)
C7	0.0312 (17)	0.044 (2)	0.0388 (18)	−0.0023 (15)	0.0052 (14)	−0.0047 (15)
C8	0.0326 (17)	0.047 (2)	0.0402 (18)	0.0097 (15)	0.0033 (14)	−0.0001 (15)
C9	0.0288 (16)	0.040 (2)	0.0440 (18)	0.0043 (15)	0.0084 (14)	0.0035 (15)
C10	0.045 (2)	0.058 (3)	0.072 (3)	−0.0053 (19)	0.018 (2)	0.005 (2)
C11	0.040 (2)	0.061 (3)	0.059 (2)	0.0173 (18)	0.0116 (18)	0.0098 (18)
C12	0.0349 (18)	0.044 (2)	0.0471 (19)	0.0047 (15)	0.0181 (15)	0.0007 (15)
C13	0.0391 (19)	0.040 (2)	0.0436 (19)	0.0139 (16)	0.0147 (15)	0.0021 (15)
C14	0.0411 (19)	0.037 (2)	0.0355 (17)	0.0069 (15)	0.0133 (14)	0.0013 (14)
C15	0.056 (2)	0.039 (2)	0.0437 (19)	0.0082 (18)	0.0229 (17)	0.0009 (15)
C16	0.066 (2)	0.031 (2)	0.043 (2)	0.0006 (18)	0.0229 (18)	0.0007 (15)
C17	0.050 (2)	0.044 (2)	0.0422 (19)	−0.0065 (18)	0.0185 (17)	0.0040 (16)
C18	0.0389 (18)	0.037 (2)	0.0357 (17)	−0.0004 (15)	0.0140 (14)	0.0037 (14)
C19	0.0384 (18)	0.035 (2)	0.0268 (15)	−0.0007 (15)	0.0079 (13)	0.0023 (13)
C20	0.0426 (19)	0.033 (2)	0.0313 (16)	0.0001 (16)	0.0034 (14)	−0.0013 (13)
C21	0.051 (2)	0.046 (2)	0.0382 (19)	0.0040 (18)	0.0054 (16)	−0.0027 (15)
C22	0.056 (2)	0.043 (2)	0.051 (2)	0.0109 (19)	−0.0056 (18)	−0.0063 (17)
C23	0.071 (3)	0.031 (2)	0.050 (2)	0.005 (2)	−0.0106 (19)	−0.0007 (16)
C24	0.055 (2)	0.040 (2)	0.051 (2)	−0.0087 (19)	−0.0022 (18)	0.0010 (17)
C25	0.045 (2)	0.032 (2)	0.0402 (18)	−0.0013 (16)	0.0014 (15)	0.0018 (14)
C26	0.043 (2)	0.047 (2)	0.0417 (19)	−0.0123 (17)	0.0088 (16)	0.0069 (16)
C27	0.0412 (19)	0.052 (2)	0.048 (2)	−0.0075 (17)	0.0199 (16)	0.0026 (16)
C28	0.0308 (18)	0.059 (2)	0.051 (2)	−0.0081 (17)	0.0102 (16)	0.0018 (17)
C29	0.053 (2)	0.090 (3)	0.076 (3)	−0.040 (2)	0.007 (2)	0.003 (2)
C30	0.052 (3)	0.103 (4)	0.084 (3)	0.020 (3)	0.024 (2)	0.016 (3)
C31	0.0327 (17)	0.049 (2)	0.0382 (17)	−0.0117 (15)	−0.0002 (14)	0.0036 (15)
C32	0.0287 (17)	0.045 (2)	0.0330 (17)	0.0011 (15)	0.0004 (13)	0.0071 (14)
C33	0.0343 (17)	0.0305 (19)	0.0387 (17)	−0.0021 (14)	0.0055 (14)	0.0041 (14)
C34	0.041 (2)	0.040 (2)	0.059 (2)	0.0050 (17)	0.0104 (17)	0.0098 (17)
C35	0.053 (2)	0.030 (2)	0.080 (3)	0.0038 (18)	0.021 (2)	0.0100 (18)
C36	0.048 (2)	0.033 (2)	0.065 (2)	−0.0106 (17)	0.0201 (18)	0.0017 (17)
C37	0.0333 (17)	0.035 (2)	0.0446 (19)	−0.0057 (15)	0.0107 (14)	0.0025 (14)
C38	0.0348 (17)	0.0320 (19)	0.0283 (15)	−0.0028 (14)	0.0088 (13)	0.0024 (13)
Cl1	0.0421 (5)	0.0607 (7)	0.0841 (7)	0.0169 (5)	0.0028 (5)	−0.0093 (5)
Cl2	0.1053 (9)	0.0366 (6)	0.0784 (7)	0.0052 (6)	−0.0020 (6)	−0.0121 (5)
Cl3	0.1106 (9)	0.0335 (6)	0.0812 (7)	−0.0052 (5)	0.0462 (7)	−0.0003 (5)
Cl4	0.0425 (5)	0.0487 (6)	0.0673 (6)	0.0013 (4)	0.0263 (4)	0.0076 (4)
Cl5	0.0520 (6)	0.0665 (7)	0.0836 (7)	0.0029 (5)	0.0250 (5)	−0.0117 (5)
Cl6	0.0946 (8)	0.0346 (6)	0.0963 (8)	0.0055 (5)	−0.0249 (6)	−0.0073 (5)
Cl7	0.0756 (8)	0.0339 (6)	0.1689 (13)	0.0078 (5)	0.0255 (8)	0.0200 (7)
Cl8	0.0348 (4)	0.0470 (6)	0.0724 (6)	−0.0119 (4)	0.0056 (4)	0.0018 (4)
N1	0.0289 (14)	0.0362 (17)	0.0358 (14)	0.0042 (12)	0.0052 (11)	−0.0002 (12)
N2	0.0303 (14)	0.0371 (17)	0.0380 (14)	0.0051 (12)	0.0089 (11)	−0.0009 (12)
N3	0.0364 (15)	0.0377 (18)	0.0385 (15)	−0.0051 (13)	0.0098 (12)	0.0044 (12)
N4	0.0294 (14)	0.0387 (17)	0.0319 (13)	−0.0072 (12)	0.0027 (11)	0.0028 (12)
O1	0.0320 (12)	0.0340 (13)	0.0526 (13)	0.0054 (10)	0.0047 (10)	−0.0073 (10)
O2	0.0340 (12)	0.0317 (13)	0.0494 (13)	0.0011 (10)	0.0156 (10)	−0.0016 (10)
O3	0.0379 (12)	0.0333 (14)	0.0517 (13)	−0.0013 (10)	0.0130 (11)	0.0013 (10)
O4	0.0297 (11)	0.0312 (13)	0.0458 (12)	−0.0035 (10)	0.0046 (9)	0.0051 (10)
O1W	0.0358 (12)	0.0373 (13)	0.0398 (12)	0.0032 (10)	0.0040 (9)	0.0017 (10)
O2W	0.0373 (12)	0.0363 (13)	0.0410 (12)	−0.0021 (10)	0.0056 (10)	0.0001 (10)
Zn1	0.02780 (18)	0.0338 (2)	0.0398 (2)	0.00256 (16)	0.00654 (15)	−0.00178 (16)
Zn2	0.03034 (19)	0.0328 (2)	0.0374 (2)	−0.00331 (16)	0.00381 (15)	0.00323 (16)

### Geometric parameters (Å, º)

**Table:**

C1—O1	1.293 (3)	C23—C24	1.366 (5)
C1—C2	1.418 (4)	C23—Cl6	1.744 (4)
C1—C6	1.422 (4)	C24—C25	1.403 (5)
C2—C3	1.361 (5)	C24—H24	0.9300
C2—Cl1	1.729 (3)	C25—C26	1.437 (5)
C3—C4	1.382 (5)	C26—N3	1.273 (4)
C3—H3	0.9300	C26—H26	0.9300
C4—C5	1.353 (5)	C27—N3	1.450 (4)
C4—Cl2	1.736 (3)	C27—C28	1.544 (4)
C5—C6	1.395 (4)	C27—H27A	0.9700
C5—H5	0.9300	C27—H27B	0.9700
C6—C7	1.442 (4)	C28—C30	1.515 (5)
C7—N1	1.271 (4)	C28—C31	1.525 (5)
C7—H7	0.9300	C28—C29	1.535 (5)
C8—N1	1.464 (3)	C29—H29A	0.9600
C8—C9	1.530 (4)	C29—H29B	0.9600
C8—H8A	0.9700	C29—H29C	0.9600
C8—H8B	0.9700	C30—H30A	0.9600
C9—C10	1.526 (4)	C30—H30B	0.9600
C9—C11	1.527 (4)	C30—H30C	0.9600
C9—C12	1.529 (4)	C31—N4	1.467 (4)
C10—H10A	0.9600	C31—H31A	0.9700
C10—H10B	0.9600	C31—H31B	0.9700
C10—H10C	0.9600	C32—N4	1.270 (4)
C11—H11A	0.9600	C32—C33	1.437 (4)
C11—H11B	0.9600	C32—H32	0.9300
C11—H11C	0.9600	C33—C34	1.394 (4)
C12—N2	1.457 (4)	C33—C38	1.423 (4)
C12—H12A	0.9700	C34—C35	1.358 (5)
C12—H12B	0.9700	C34—H34A	0.9300
C13—N2	1.269 (4)	C35—C36	1.379 (5)
C13—C14	1.449 (4)	C35—Cl7	1.737 (3)
C13—H13	0.9300	C36—C37	1.361 (4)
C14—C15	1.400 (4)	C36—H36	0.9300
C14—C19	1.419 (4)	C37—C38	1.413 (4)
C15—C16	1.356 (5)	C37—Cl8	1.726 (3)
C15—H15	0.9300	C38—O4	1.290 (3)
C16—C17	1.369 (5)	N1—Zn1	2.044 (2)
C16—Cl3	1.738 (3)	N2—Zn1	2.103 (2)
C17—C18	1.375 (4)	N3—Zn2	2.092 (3)
C17—H17	0.9300	N4—Zn2	2.061 (2)
C18—C19	1.407 (4)	O1—Zn1	2.004 (2)
C18—Cl4	1.733 (3)	O2—Zn1	1.960 (2)
C19—O2	1.302 (3)	O3—Zn2	1.972 (2)
C20—O3	1.302 (3)	O4—Zn2	2.031 (2)
C20—C25	1.412 (4)	O1W—Zn1	2.0651 (19)
C20—C21	1.417 (4)	O1W—H1W1	0.8866
C21—C22	1.367 (5)	O1W—H2W1	0.8928
C21—Cl5	1.727 (4)	O2W—Zn2	2.053 (2)
C22—C23	1.367 (5)	O2W—H1W2	0.8843
C22—H22	0.9300	O2W—H2W2	0.8903
			
O1—C1—C2	121.1 (3)	N3—C27—C28	112.2 (3)
O1—C1—C6	124.1 (3)	N3—C27—H27A	109.2
C2—C1—C6	114.8 (3)	C28—C27—H27A	109.2
C3—C2—C1	123.5 (3)	N3—C27—H27B	109.2
C3—C2—Cl1	118.9 (3)	C28—C27—H27B	109.2
C1—C2—Cl1	117.6 (3)	H27A—C27—H27B	107.9
C2—C3—C4	119.6 (3)	C30—C28—C31	111.2 (3)
C2—C3—H3	120.2	C30—C28—C29	110.6 (3)
C4—C3—H3	120.2	C31—C28—C29	105.3 (3)
C5—C4—C3	119.9 (3)	C30—C28—C27	108.2 (3)
C5—C4—Cl2	120.5 (3)	C31—C28—C27	111.8 (3)
C3—C4—Cl2	119.5 (3)	C29—C28—C27	109.7 (3)
C4—C5—C6	121.5 (3)	C28—C29—H29A	109.5
C4—C5—H5	119.3	C28—C29—H29B	109.5
C6—C5—H5	119.3	H29A—C29—H29B	109.5
C5—C6—C1	120.6 (3)	C28—C29—H29C	109.5
C5—C6—C7	115.8 (3)	H29A—C29—H29C	109.5
C1—C6—C7	123.3 (3)	H29B—C29—H29C	109.5
N1—C7—C6	127.6 (3)	C28—C30—H30A	109.5
N1—C7—H7	116.2	C28—C30—H30B	109.5
C6—C7—H7	116.2	H30A—C30—H30B	109.5
N1—C8—C9	115.7 (2)	C28—C30—H30C	109.5
N1—C8—H8A	108.4	H30A—C30—H30C	109.5
C9—C8—H8A	108.4	H30B—C30—H30C	109.5
N1—C8—H8B	108.4	N4—C31—C28	114.6 (3)
C9—C8—H8B	108.4	N4—C31—H31A	108.6
H8A—C8—H8B	107.4	C28—C31—H31A	108.6
C10—C9—C11	110.3 (3)	N4—C31—H31B	108.6
C10—C9—C12	108.1 (3)	C28—C31—H31B	108.6
C11—C9—C12	110.0 (3)	H31A—C31—H31B	107.6
C10—C9—C8	110.7 (3)	N4—C32—C33	126.9 (3)
C11—C9—C8	105.9 (3)	N4—C32—H32	116.6
C12—C9—C8	111.9 (2)	C33—C32—H32	116.6
C9—C10—H10A	109.5	C34—C33—C38	120.3 (3)
C9—C10—H10B	109.5	C34—C33—C32	116.4 (3)
H10A—C10—H10B	109.5	C38—C33—C32	123.2 (3)
C9—C10—H10C	109.5	C35—C34—C33	121.1 (3)
H10A—C10—H10C	109.5	C35—C34—H34A	119.4
H10B—C10—H10C	109.5	C33—C34—H34A	119.4
C9—C11—H11A	109.5	C34—C35—C36	120.3 (3)
C9—C11—H11B	109.5	C34—C35—Cl7	120.3 (3)
H11A—C11—H11B	109.5	C36—C35—Cl7	119.3 (3)
C9—C11—H11C	109.5	C37—C36—C35	119.5 (3)
H11A—C11—H11C	109.5	C37—C36—H36	120.3
H11B—C11—H11C	109.5	C35—C36—H36	120.3
N2—C12—C9	112.7 (3)	C36—C37—C38	123.3 (3)
N2—C12—H12A	109.1	C36—C37—Cl8	118.5 (2)
C9—C12—H12A	109.1	C38—C37—Cl8	118.2 (2)
N2—C12—H12B	109.1	O4—C38—C37	120.7 (3)
C9—C12—H12B	109.1	O4—C38—C33	123.9 (3)
H12A—C12—H12B	107.8	C37—C38—C33	115.4 (3)
N2—C13—C14	125.4 (3)	C7—N1—C8	118.1 (3)
N2—C13—H13	117.3	C7—N1—Zn1	124.7 (2)
C14—C13—H13	117.3	C8—N1—Zn1	116.7 (2)
C15—C14—C19	120.0 (3)	C13—N2—C12	120.8 (3)
C15—C14—C13	116.8 (3)	C13—N2—Zn1	126.3 (2)
C19—C14—C13	123.1 (3)	C12—N2—Zn1	112.3 (2)
C16—C15—C14	121.2 (3)	C26—N3—C27	120.3 (3)
C16—C15—H15	119.4	C26—N3—Zn2	126.4 (2)
C14—C15—H15	119.4	C27—N3—Zn2	113.3 (2)
C15—C16—C17	120.9 (3)	C32—N4—C31	118.6 (3)
C15—C16—Cl3	120.2 (3)	C32—N4—Zn2	124.3 (2)
C17—C16—Cl3	118.9 (3)	C31—N4—Zn2	117.1 (2)
C16—C17—C18	118.5 (3)	C1—O1—Zn1	128.29 (19)
C16—C17—H17	120.8	C19—O2—Zn1	129.3 (2)
C18—C17—H17	120.8	C20—O3—Zn2	130.0 (2)
C17—C18—C19	123.9 (3)	C38—O4—Zn2	125.89 (19)
C17—C18—Cl4	119.1 (3)	Zn1—O1W—H1W1	105.9
C19—C18—Cl4	116.9 (2)	Zn1—O1W—H2W1	108.6
O2—C19—C18	119.1 (3)	H1W1—O1W—H2W1	108.1
O2—C19—C14	125.6 (3)	Zn2—O2W—H1W2	107.1
C18—C19—C14	115.3 (3)	Zn2—O2W—H2W2	112.3
O3—C20—C25	125.3 (3)	H1W2—O2W—H2W2	113.0
O3—C20—C21	119.5 (3)	O2—Zn1—O1	94.56 (9)
C25—C20—C21	115.2 (3)	O2—Zn1—N1	130.35 (9)
C22—C21—C20	123.9 (3)	O1—Zn1—N1	90.04 (9)
C22—C21—Cl5	118.3 (3)	O2—Zn1—O1W	105.90 (8)
C20—C21—Cl5	117.8 (3)	O1—Zn1—O1W	94.60 (8)
C21—C22—C23	119.0 (4)	N1—Zn1—O1W	122.98 (9)
C21—C22—H22	120.5	O2—Zn1—N2	88.53 (9)
C23—C22—H22	120.5	O1—Zn1—N2	176.45 (9)
C24—C23—C22	120.6 (3)	N1—Zn1—N2	86.62 (10)
C24—C23—Cl6	119.3 (3)	O1W—Zn1—N2	86.21 (9)
C22—C23—Cl6	120.1 (3)	O3—Zn2—O4	96.47 (9)
C23—C24—C25	120.9 (4)	O3—Zn2—O2W	110.58 (8)
C23—C24—H24	119.5	O4—Zn2—O2W	89.35 (8)
C25—C24—H24	119.5	O3—Zn2—N4	136.04 (9)
C24—C25—C20	120.4 (3)	O4—Zn2—N4	87.78 (9)
C24—C25—C26	116.6 (3)	O2W—Zn2—N4	113.21 (9)
C20—C25—C26	123.0 (3)	O3—Zn2—N3	88.45 (10)
N3—C26—C25	126.4 (3)	O4—Zn2—N3	173.38 (9)
N3—C26—H26	116.8	O2W—Zn2—N3	93.08 (9)
C25—C26—H26	116.8	N4—Zn2—N3	85.60 (10)

### Hydrogen-bond geometry (Å, º)

**Table:**

*D*—H···*A*	*D*—H	H···*A*	*D*···*A*	*D*—H···*A*
O1*W*—H1*W*1···Cl5^i^	0.89	2.76	3.472 (2)	139
O1*W*—H1*W*1···O3^i^	0.89	2.05	2.825 (3)	145
O1*W*—H2*W*1···Cl8^i^	0.89	2.62	3.235 (2)	127
O1*W*—H2*W*1···O4^i^	0.89	1.86	2.681 (3)	153
O2*W*—H1*W*2···Cl4^ii^	0.88	2.51	3.226 (2)	139
O2*W*—H1*W*2···O2^ii^	0.88	2.04	2.807 (3)	144
O2*W*—H2*W*2···Cl1^ii^	0.89	2.62	3.340 (2)	139
O2*W*—H2*W*2···O1^ii^	0.89	2.01	2.749 (3)	140
C8—H8*A*···O4^iii^	0.97	2.56	3.310 (4)	134

Symmetry codes: (i) *x*−1, *y*, *z*; (ii) *x*+1, *y*, *z*; (iii) *x*−1/2, −*y*+3/2, *z*+1/2.

## References

[bb1] Allen, F. H., Kennard, O., Watson, D. G., Brammer, L., Orpen, A. G. & Taylor, R. (1987). *J. Chem. Soc. Perkin Trans. 2*, pp. S1–19.

[bb2] Bernstein, J., Davis, R. E., Shimoni, L. & Chang, N.-L. (1995). *Angew. Chem. Int. Ed. Engl.* **34**, 1555–1573.

[bb3] Blower, P. J. (1998). *Transition Met. Chem.*, **23**, 109–112.

[bb4] Bondi, A. (1964). *J. Phys. Chem.* **68**, 441-452.

[bb5] Bruker (2005). *APEX2*, *SAINT* and *SADABS* Bruker AXS Inc., Madison, Wisconsin, USA.

[bb6] Granovski, A. D., Nivorozhkin, A. L. & Minkin, V. I. (1993). *Coord. Chem. Rev* **126**, 1–69.

[bb7] Sheldrick, G. M. (2008). *Acta Cryst.* A**64**, 112–122. 10.1107/S0108767307043930 18156677

[bb8] Spek, A. L. (2009). *Acta Cryst.* D**65**, 148–155. 10.1107/S090744490804362X PMC2631630 19171970

[bb9] Zhong-Lu, Y., Xiao, H., Jia, W. & Jing-Yun, C. (2006). *Jiegou Huaxue (Chin. J. Struct. Chem.)* **25**, 1043–1047.

